# Supplementary material for: Use of epigenetically modified bacteriophage and dual beta-lactams to treat a Mycobacterium abscessus sternal wound infection
Source: Nat Commun. 2024 Nov 28;15:10360. doi: 10.1038/s41467-024-54666-4 (PMC11604996; doi:10.1038/s41467-024-54666-4)
Supplement: Supplementary file 1 — Supplementary Information [file 41467_2024_54666_MOESM1_ESM.pdf]

Table S1. Plasmids present in DCC7 strains

	Genome Status	# Plasmids (size)	Larger plasmid similarity to pGD276A-1	Smaller plasmid similarity to pGD276A-2
GD276A (R)	Complete	2 (93 kb, 41 kb)	Self	Self
GD276B (S)	Complete	2 (93 kb, 41 kb)	Identical	Identical
GD272 (R)	Complete	2 (93 kb, 18 kb)	Identical	18 kb identical[2]
GD01 (R)	Complete	0	Not present	Not present
GD10 (R)	WGS	1 (41 kb[1])	Not present	Identical
GD30 (R)	WGS	1 (41 kb[1])	Not present	Identical
GD58 (R)	WGS	2 (93 kb, 41 kb[1])	Identical	Identical
GD82 (R)	Complete	0	Not present	Not present
GD104 (R)	WGS	1 (18 kb[1])	Not present	18 kb identical[2]
GD262 (R)	WGS	1 (41 kb[1])	Not present	Identical
GD273 (R)	WGS	1 (41 kb[1])	Not present	Identical

[1]These plasmids, though present, were split into more than one contig in their respective WGS assemblies. To assess similarity to the GD276A plasmids, relevant contigs were joined.

[2]Some genomes contain a smaller version of this plasmid which is only ~18 kb instead of ~41 kb. In the larger 41 kb version, the entire 18 kb plasmid is present and is flanked by a ~2.1 kb repeated sequence. There is also ~21 kb of sequence that is unique to the 41 kb version.

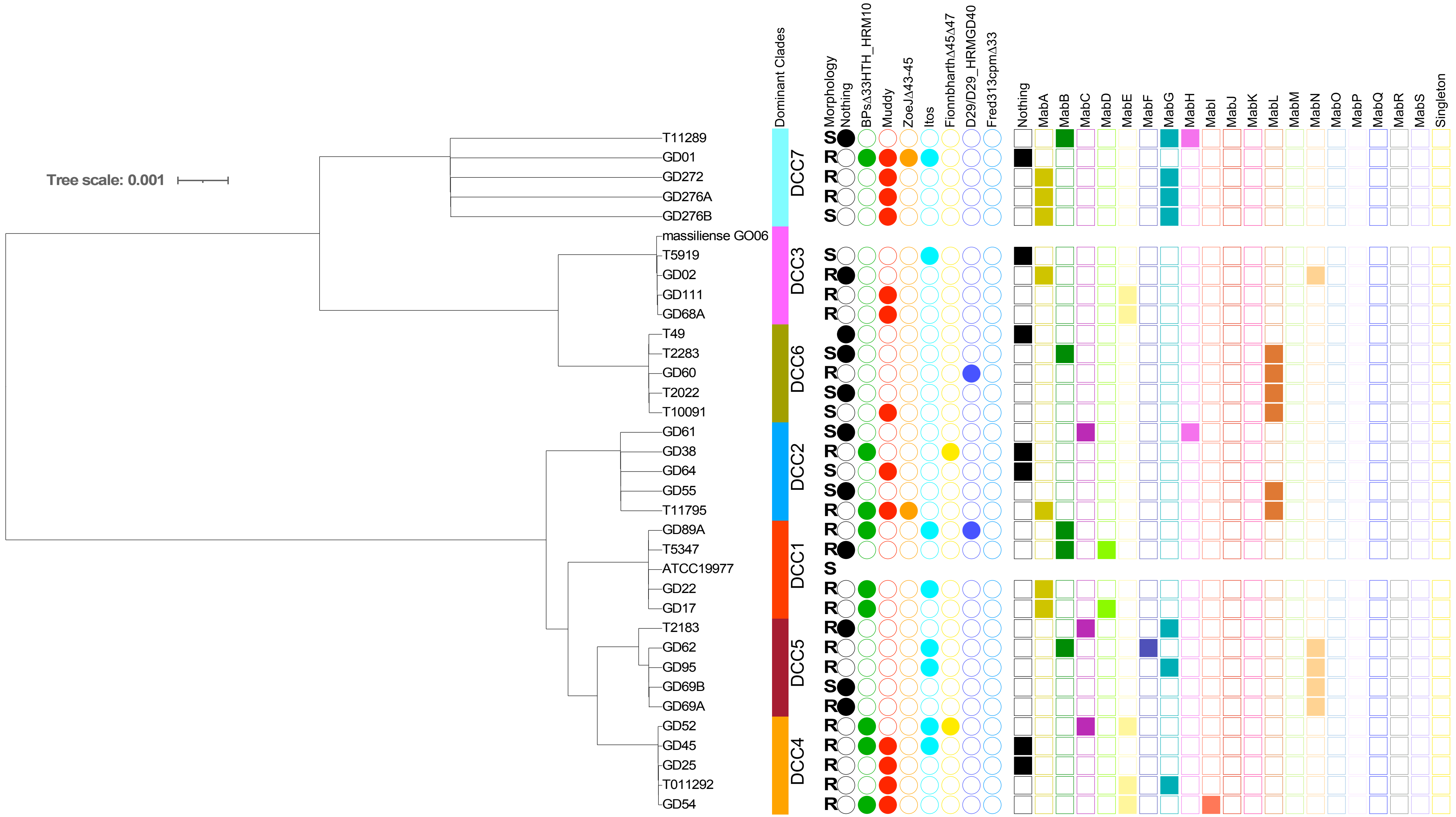

Figure S1. Phage susceptibilities and prophage content of *M. abscessus* strains. A phylogeny of *M. abscessus* strains (as in Fig. 1) representing the major groups of dominating circulating clones (DCC1-DCC7) is shown together with their phage susceptibility profiles, with filled circles indicating susceptibility (with an efficiency of plaquing of great than 0.1 relative to *M. smegmatis*). The presence of a resident prophage in each strain is indicated by filled squares, with prophage classed within their genome cluster (MabA, MabB etc) as described previously[1, 2].

1. Dedrick RM, *et al*. The Prophage and Plasmid Mobilome as a Likely Driver of Mycobacterium abscessus Diversity. *mBio* **12**, (2021).

2. Dedrick RM, *et al*. Mycobacterium abscessus Strain Morphotype Determines Phage Susceptibility, the Repertoire of Therapeutically Useful Phages, and Phage Resistance. *mBio* **12**, (2021).

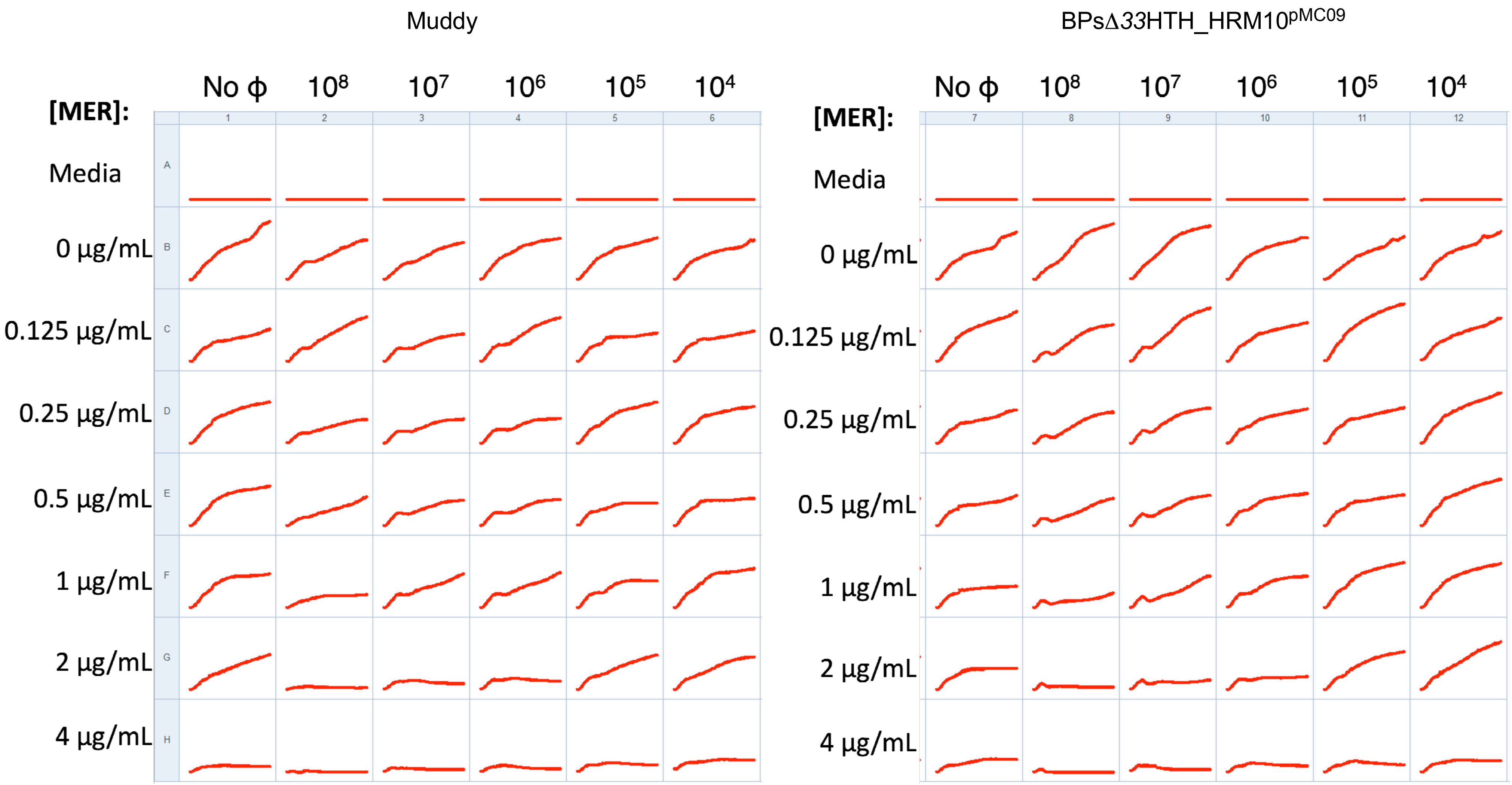

Figure S2. Phage-antibiotic synergy. *M. abscessus* GD276B (2 x 10^7 CFU) cells were incubated with antibiotics and phages Muddy (left panel) or BPsΔ 33HTH_HRM10^{pMC09} (right panel) and the OD_{600} measured for 60 hours. The first rows of each panel contain no bacteria, phage or antibiotics, the second row contains no meropenem, and the rows below contain meropenem at the indicated concentrations. The first columns contain no phage, and the five columns to the right contain either phage Muddy at 1.4 x 10^8, 1.4 x 10^7, 1.4 x 10^6, 1.4 x 10^5, and 1.4 x 10^4 PFU, respectively, or phage BPsΔ 33HTH_HRM10^{pMC09} at 3.2 x 10^8, 3.2 x 10^7, 3.2 x 10^6, 3.2 x 10^5, and 3.2 x 10^4 PFU, respectively. All samples containing meropenem also contain avibactam (4 µg/ml). Data are provided in the Source Data File.
