# Supplementary material for: Use of epigenetically modified bacteriophage and dual beta-lactams to treat a Mycobacterium abscessus sternal wound infection
Source: Nat Commun. 2024 Nov 28;15:10360. doi: 10.1038/s41467-024-54666-4 (PMC11604996; doi:10.1038/s41467-024-54666-4)
Supplement: Supplementary file 3 — Reporting Summary [file 41467_2024_54666_MOESM3_ESM.pdf]

nature portfolio

Corresponding author(s): Barry N. Kreiswirth, Graham F. Hatfull, Anne Friedland

Last updated by author(s): Nov 2, 2024

Reporting Summary

Nature Portfolio wishes to improve the reproducibility of the work that we publish. This form provides structure for consistency and transparency in reporting. For further information on Nature Portfolio policies, see our Editorial Policies and the Editorial Policy Checklist.

Statistics

For all statistical analyses, confirm that the following items are present in the figure legend, table legend, main text, or Methods section.

n/a	Confirmed	
☐	☒	The exact sample size (*n*) for each experimental group/condition, given as a discrete number and unit of measurement
☐	☒	A statement on whether measurements were taken from distinct samples or whether the same sample was measured repeatedly
☒	☐	The statistical test(s) used AND whether they are one- or two-sided<br>*Only common tests should be described solely by name; describe more complex techniques in the Methods section.*
☒	☐	A description of all covariates tested
☒	☐	A description of any assumptions or corrections, such as tests of normality and adjustment for multiple comparisons
☒	☐	A full description of the statistical parameters including central tendency (e.g. means) or other basic estimates (e.g. regression coefficient) AND variation (e.g. standard deviation) or associated estimates of uncertainty (e.g. confidence intervals)
☒	☐	For null hypothesis testing, the test statistic (e.g. *F*, *t*, *r*) with confidence intervals, effect sizes, degrees of freedom and *P* value noted<br>*Give P values as exact values whenever suitable.*
☒	☐	For Bayesian analysis, information on the choice of priors and Markov chain Monte Carlo settings
☒	☐	For hierarchical and complex designs, identification of the appropriate level for tests and full reporting of outcomes
☒	☐	Estimates of effect sizes (e.g. Cohen's *d*, Pearson's *r*), indicating how they were calculated

Our web collection on statistics for biologists contains articles on many of the points above.

Software and code

Policy information about availability of computer code

Data collection	BioTek Gen5 Data Analysis Software v 3.10
Data analysis	GraphPad Prism version 10.0

For manuscripts utilizing custom algorithms or software that are central to the research but not yet described in published literature, software must be made available to editors and reviewers. We strongly encourage code deposition in a community repository (e.g. GitHub). See the Nature Portfolio guidelines for submitting code & software for further information.

Data

Policy information about availability of data

All manuscripts must include a data availability statement. This statement should provide the following information, where applicable:

- Accession codes, unique identifiers, or web links for publicly available datasets
- A description of any restrictions on data availability
- For clinical datasets or third party data, please ensure that the statement adheres to our policy

Data Availability. All datasets and materials generated during and/or analyzed during the current study are available from the corresponding author on request without restriction except for clinical details due to privacy laws. The source data used for Fig. 6C are provided with his paper as 'Source Data File–Synergy_Experiment' and all raw images are provided as 'Source Data File–Raw_Images'. GenBank accession numbers of bacterial genomes are as follows: M. abscessus GD272 [https://www.ncbi.nlm.nih.gov/nuccore/CP167809] and its plasmids pGD272-1 [https://www.ncbi.nlm.nih.gov/nuccore/CP167810] and pGD272-2

[https://www.ncbi.nlm.nih.gov/nuccore/CP167811]; GD276A [https://www.ncbi.nlm.nih.gov/nuccore/CP167806] and its plasmids pGD276A-1 [https://www.ncbi.nlm.nih.gov/nuccore/CP167807] and pGD276A-2 [https://www.ncbi.nlm.nih.gov/nuccore/CP167808]; GD276B [https://www.ncbi.nlm.nih.gov/nuccore/CP167803] and its plasmids pGD276B-1 [https://www.ncbi.nlm.nih.gov/nuccore/CP167804] and pGD276B-2 [https://www.ncbi.nlm.nih.gov/nuccore/CP167805]; GD276A_RMM3 [https://www.ncbi.nlm.nih.gov/nuccore/CP167794] and its plasmids pGD276A_RMM3-1 [https://www.ncbi.nlm.nih.gov/nuccore/CP167795] and pGD276A_RMM3-2 [https://www.ncbi.nlm.nih.gov/nuccore/CP167796]; GD276A_RMB09_3 [https://www.ncbi.nlm.nih.gov/nuccore/CP167797] and its plasmids pGD276A_RMB09_3-1 [https://www.ncbi.nlm.nih.gov/nuccore/CP167798] and pGD276A_RMB09_3-2 [https://www.ncbi.nlm.nih.gov/nuccore/CP167799]; GD276A_RMB09_1 [https://www.ncbi.nlm.nih.gov/nuccore/CP167800] and its plasmids pGD276A_RMB09_1-1 [https://www.ncbi.nlm.nih.gov/nuccore/CP167801] and pGD276A_RMB09_1-2 [https://www.ncbi.nlm.nih.gov/nuccore/CP167802]. .

Research involving human participants, their data, or biological material

Policy information about studies with human participants or human data. See also policy information about sex, gender (identity/presentation), and sexual orientation and race, ethnicity and racism.

Reporting on sex and gender	Single case only reported.
Reporting on race, ethnicity, or other socially relevant groupings	Single case only reported
Population characteristics	Single case only reported
Recruitment	Single case only reported
Ethics oversight	Approved by UNC IRB under IRB numbers 22-1710, 19-2446

Note that full information on the approval of the study protocol must also be provided in the manuscript.

Field-specific reporting

Please select the one below that is the best fit for your research. If you are not sure, read the appropriate sections before making your selection.

☒ Life sciences ☐ Behavioural & social sciences ☐ Ecological, evolutionary & environmental sciences

For a reference copy of the document with all sections, see nature.com/documents/nr-reporting-summary-flat.pdf

Life sciences study design

All studies must disclose on these points even when the disclosure is negative.

Sample size	This paper describes the treatment and outcomes of one patient. Approximately 20 phages and derivatives were screened for phage suscpetibilties.
Data exclusions	No data were excluded from the analyses.
Replication	The patient treatment outcomes are not subject to replication. All replicates of phage susceptibility profiles were consistent, including the analyses of very closely related M. abscessus strains isolated at different times. The synergy experiment shown is consistent with other experiments although we note that measurement of OD600 of Mycobacterium abscessus cultures is subject due to considerable variability due to cell cllumping, a common phenomenon of Mycobacterium cultures. Detergent could not be used due to the sensitivity of phages to Tween 80.
Randomization	Not relevant to the treatment and outcomes of a single patient.
Blinding	Not relevant to the treatment and outcomes of a single patient.

Reporting for specific materials, systems and methods

We require information from authors about some types of materials, experimental systems and methods used in many studies. Here, indicate whether each material, system or method listed is relevant to your study. If you are not sure if a list item applies to your research, read the appropriate section before selecting a response.

Materials & experimental systems

n/a	Involved in the study	
☐	☒	Antibodies
☒	☐	Eukaryotic cell lines
☒	☐	Palaeontology and archaeology
☒	☐	Animals and other organisms
☐	☒	Clinical data
☒	☐	Dual use research of concern
☒	☐	Plants

Methods

n/a	Involved in the study	
☒	☐	ChIP-seq
☒	☐	Flow cytometry
☒	☐	MRI-based neuroimaging

Antibodies

Antibodies used	Patient serum.
Validation	Not applicable.

Clinical data

Policy information about clinical studies

All manuscripts should comply with the ICMJE guidelines for publication of clinical research and a completed CONSORT checklist must be included with all submissions.

Clinical trial registration	Not a clinical trial
Study protocol	No study protocol for single case
Data collection	Data was collected from the electronic health record of the patient included to cover the period starting at the time of lung transplant which was ~5 months prior to infection and ending at the time of ~550 days after infection. Dates are not provided to protect the anonymity of the patient.
Outcomes	No pre-defined outcome measures were used

Plants

Seed stocks	*Report on the source of all seed stocks or other plant material used. If applicable, state the seed stock centre and catalogue number. If plant specimens were collected from the field, describe the collection location, date and sampling procedures.*
Novel plant genotypes	*Describe the methods by which all novel plant genotypes were produced. This includes those generated by transgenic approaches, gene editing, chemical/radiation-based mutagenesis and hybridization. For transgenic lines, describe the transformation method, the number of independent lines analyzed and the generation upon which experiments were performed. For gene-edited lines, describe the editor used, the endogenous sequence targeted for editing, the targeting guide RNA sequence (if applicable) and how the editor was applied.*
Authentication	*Describe any authentication procedures for each seed stock used or novel genotype generated. Describe any experiments used to assess the effect of a mutation and, where applicable, how potential secondary effects (e.g. second site T-DNA insertions, mosiacism, off-target gene editing) were examined.*
