# Supplementary figures and images for: Use of epigenetically modified bacteriophage and dual beta-lactams to treat a Mycobacterium abscessus sternal wound infection
Source: Nat Commun. 2024 Nov 28;15:10360. doi: 10.1038/s41467-024-54666-4 (PMC11604996; doi:10.1038/s41467-024-54666-4)

A

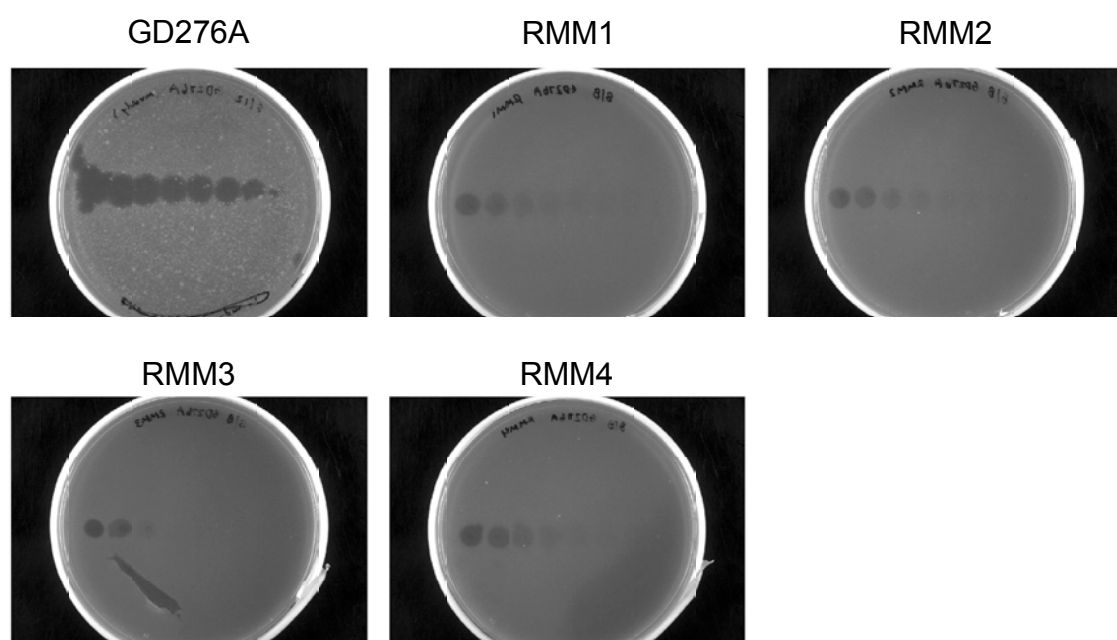

B

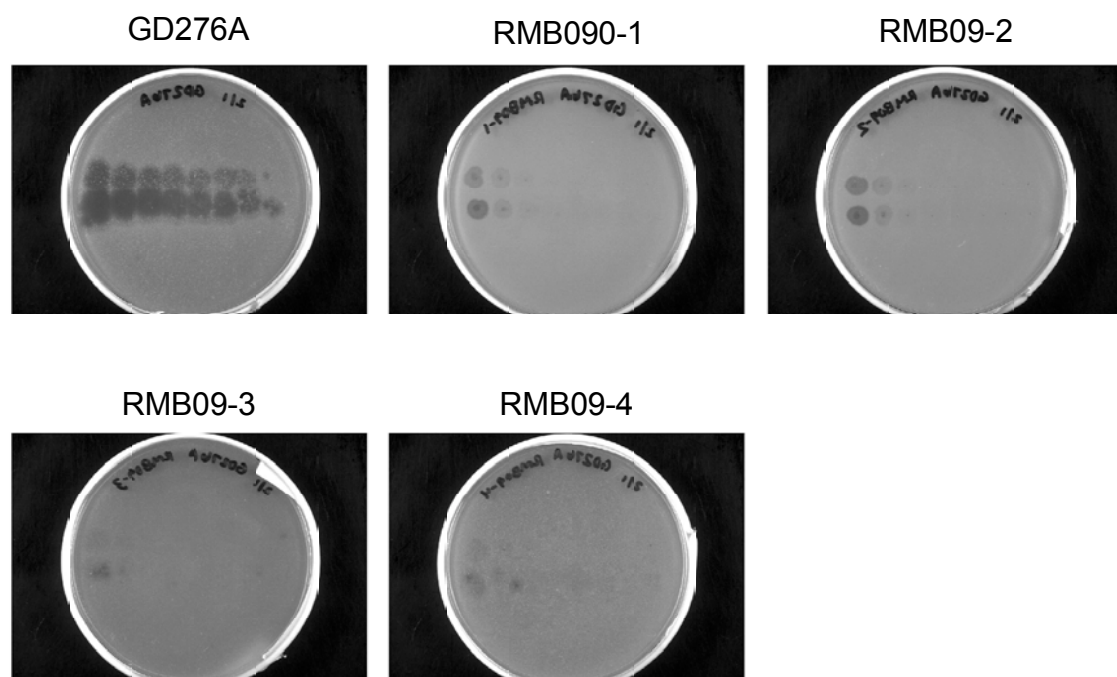

Supplement: Supplementary file 4 — Source Data [file 41467_2024_54666_MOESM4_ESM.zip › Source Data 11-16-24/Source_Data_FIg.5red.pdf]

C

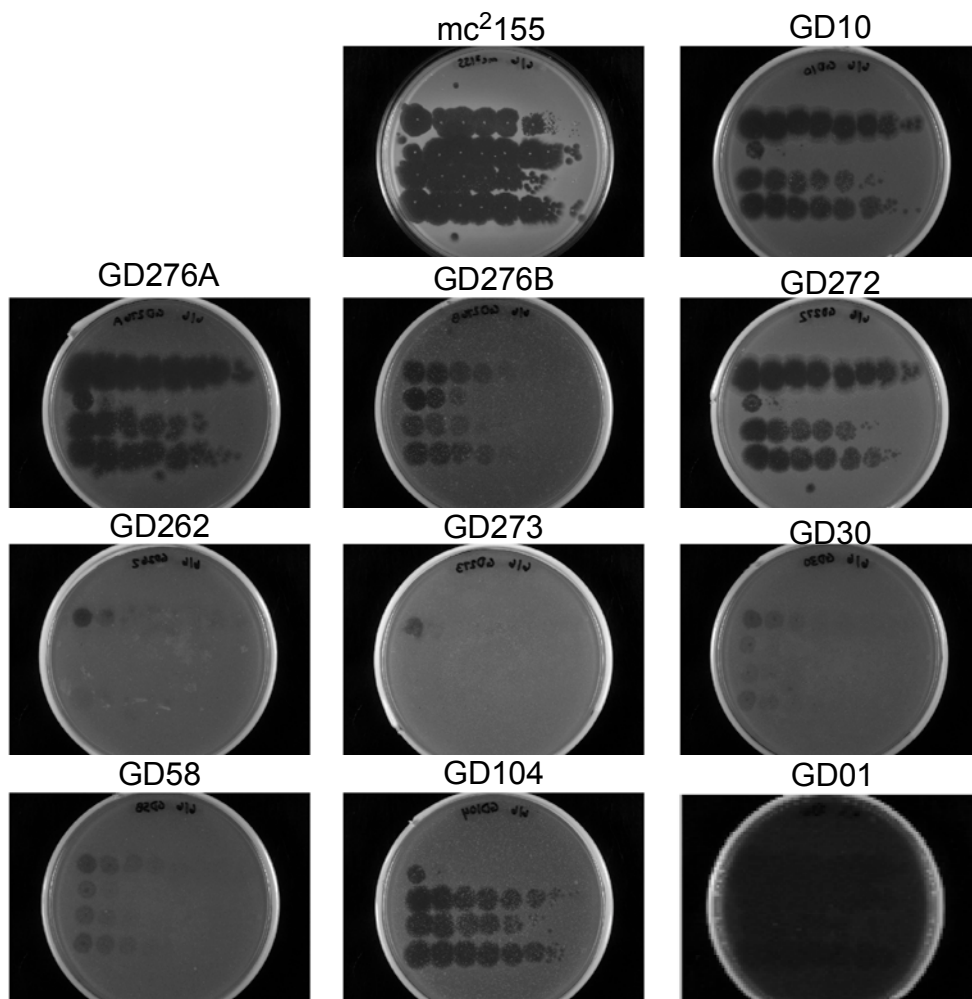

D

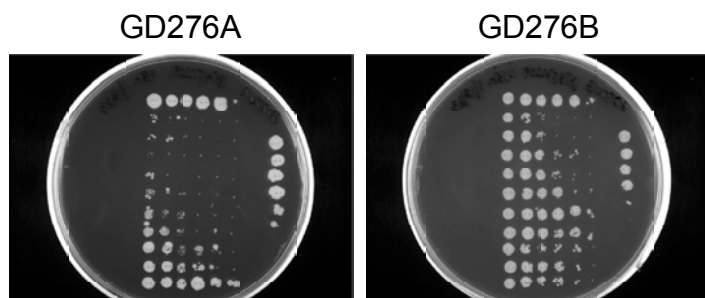

E

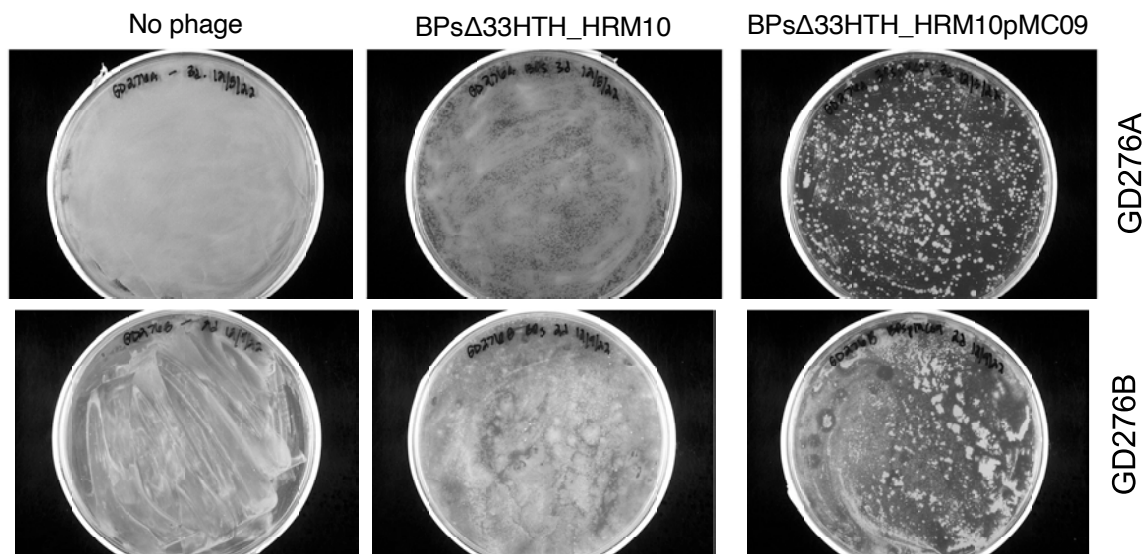

Supplement: Supplementary file 4 — Source Data [file 41467_2024_54666_MOESM4_ESM.zip › Source Data 11-16-24/Source_Data_Fig.4red.pdf]

A

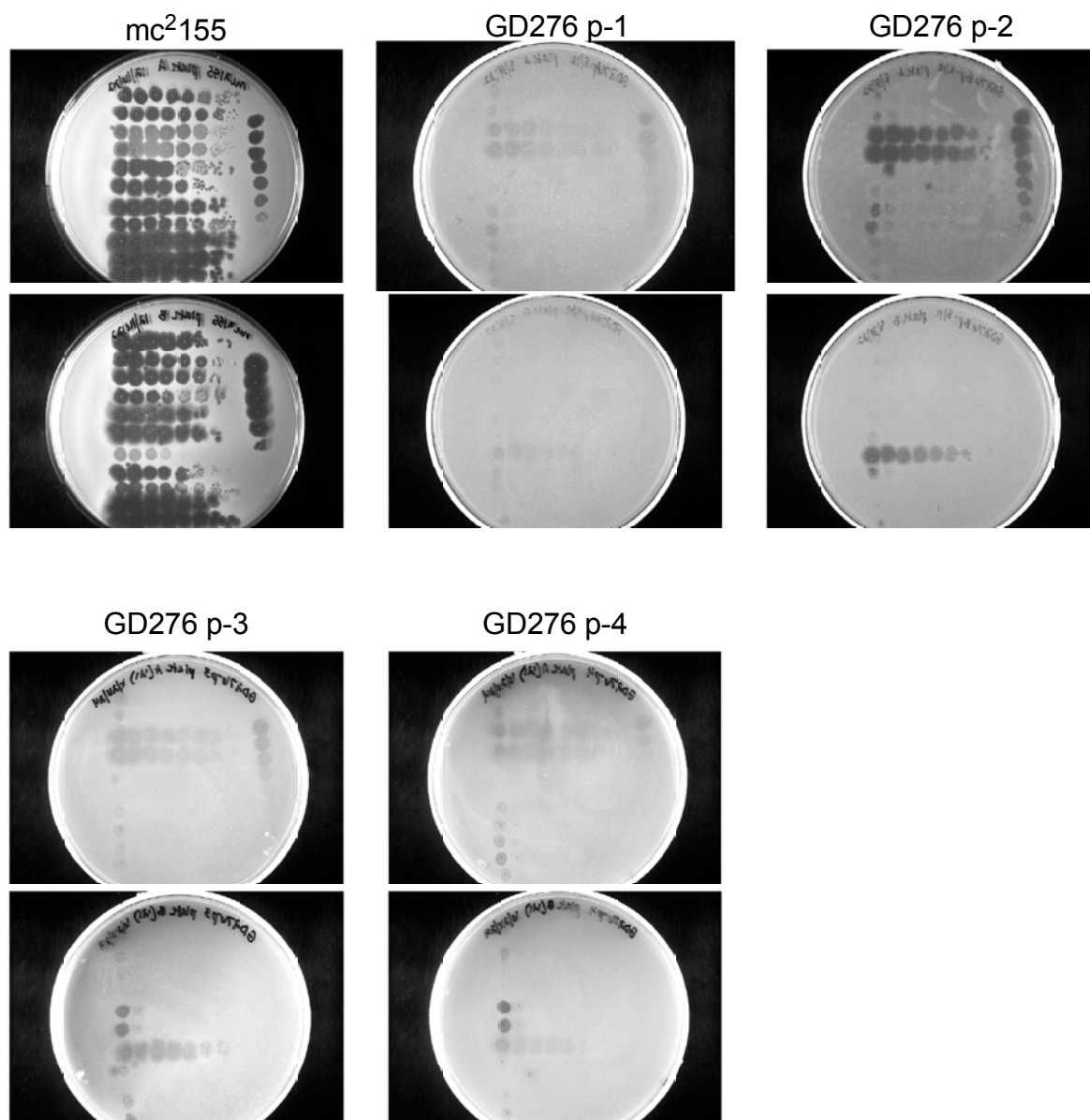

B

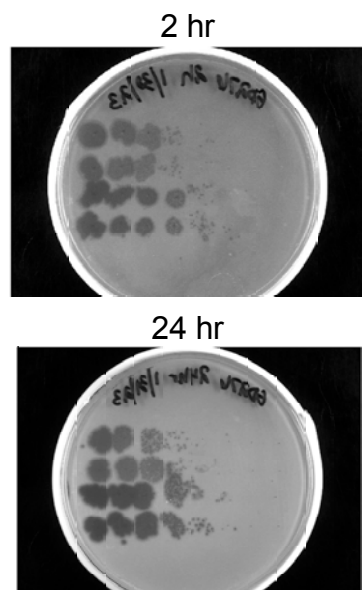

Supplement: Supplementary file 4 — Source Data [file 41467_2024_54666_MOESM4_ESM.zip › Source Data 11-16-24/Source_Data_Fig.6red.pdf]
